# Characterization of the complete mitochondrial genome of the critically endangered Southern River Terrapin, *Batagur affinis affinis* (Reptilia: Geoemydidae)

**DOI:** 10.1080/23802359.2023.2222851

**Published:** 2023-07-04

**Authors:** Mohd Hairul Mohd Salleh, Yuzine Esa, Han Ming Gan

**Affiliations:** a Department of Aquaculture, Faculty of Agriculture, Universiti Putra Malaysia, Serdang, Selangor, Malaysia; b Corporate Planning Division, Royal Malaysian Customs Department, Presint 2, Putrajaya, Malaysia; c International Institute of Aquaculture and Aquatic Sciences, Universiti Putra Malaysia, Port Dickson, Negeri Sembilan, Malaysia; d Patriot Biotech Sdn Bhd, Selangor, Malaysia

**Keywords:** Mitogenome, genes, protein-coding, phylogenetic tree, *Batagur kachuga*

## Abstract

In this study, we report the nearly complete mitochondrial sequence of *Batagur affinis affinis*. The assembled mitogenome consists of 13 PCGs, 22 tRNA genes, two rRNAs and one near-complete D-loop region. Of the annotated genes, the ND6 subunit gene and eight tRNA genes were encoded on the L-strand, while the remaining genes were dispersed on the H-strand. Except for CO1, which has a GTG start codon, all protein-coding genes begin with ATG. The mitogenome has been deposited in NCBI GenBank under the accession number OQ409915. Phylogenetic tree analysis based on publicly available mitogenomes indicate the sister grouping of *B. affinis affinis* with *B. kachuga*.

## Introduction

The Southern River Terrapin, locally known as Tuntung Sungai, is a member of the genus *Batagur*, a family of Geoemydidae and the class Reptilia. The six species in the genus *Batagur* are the Three-striped Roofed Turtle (*Batagur dhongoka*), Northern River Terrapin (*Batagur baska*), Red-crowned Roofed Turtle (*Batagur kachuga*), Burmese Roofed Turtle (*Batagur trivittata*), Southern River Terrapin (*Batagur affinis* ssp.), and Painted Terrapin (*Batagur borneoensis*) (Uwe and Havas 2007). Five of the six *Batagur* species, including the study’s focal species, are on the list of the top 25 most endangered turtles, except for *Batagur dhongoka* (Turtle Conservation Coalition 2011), which is classified as critically endangered on the IUCN 2000 Red List (Horne et al. 2016) and is close to extinction. *Batagur affinis* ssp. (Cantor 1847) is among 24 species of turtles found in Malaysia (Mohd-Salleh et al. 2022), whose closest Southeast Asian cousins are *B. baska* and *B. kachuga*.

According to Praschag et al. (2007), *B. baska* was made up of at least two heritably different species: populations of *B. affinis affinis* in the Kedah and Perak river systems on the west coast of Peninsular Malaysia. However, Salleh et al. (2022) and Salleh et al. (2023) recently stated *B. affinis edwardmolli* is also found in Kedah, Malaysia. On the other hand, the populations in the Terengganu river basin were found to be *B. affinis edwardmolli*.

## Materials and methods

**Figure 1.**
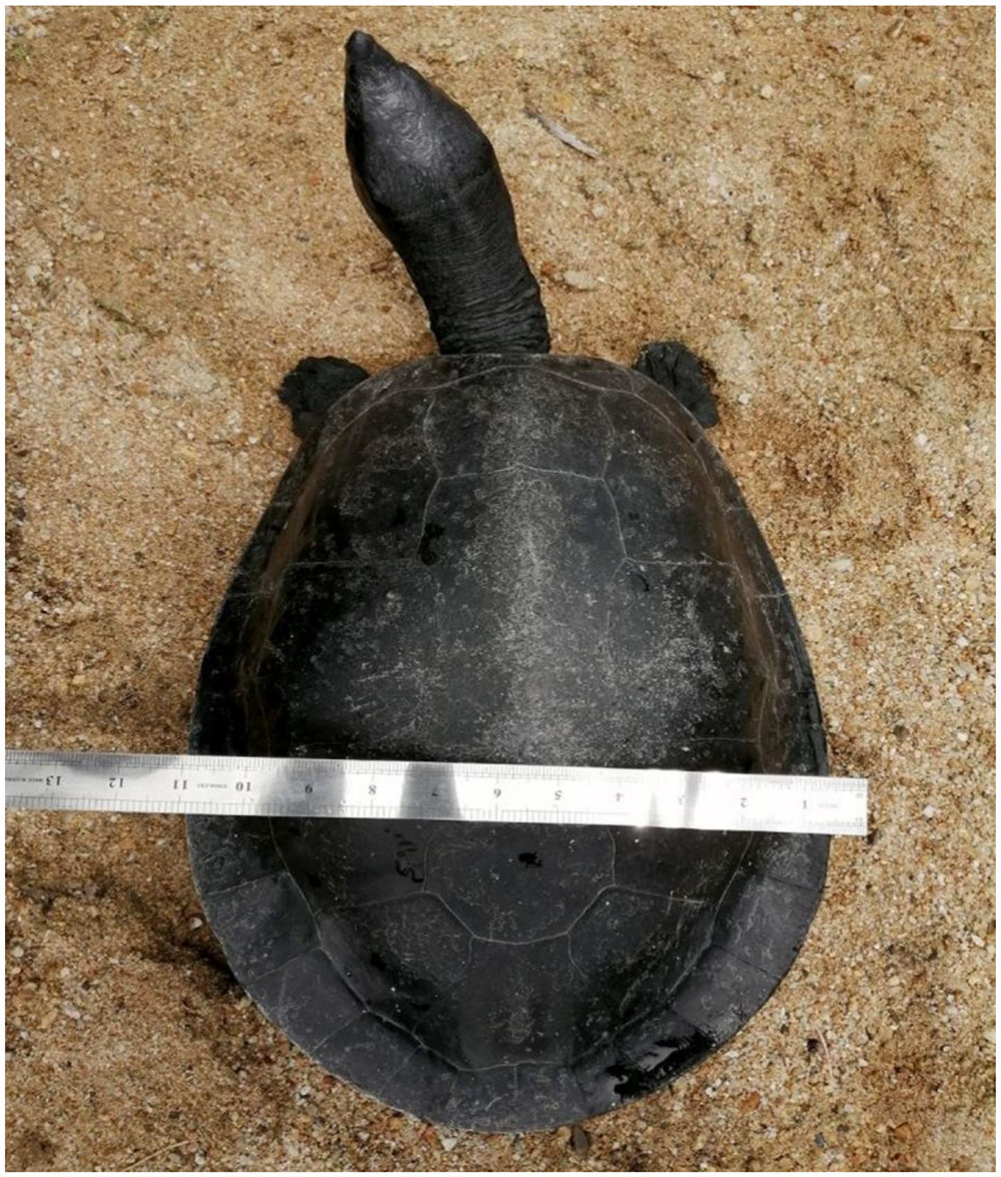
Represent an adult Southern River Terrapin (*B. affinis affinis*) from the Southern River Terrapin Breeding Center in Bota Kanan, Perak, Malaysia (own photo).

In this study, we used the genome skimming approach to recover the near-complete mitochondrial genome of *B. affinis affinis* (Figure 1). The blood sample was collected from a female individual at the Southern River Terrapin Conservation Center, Bota Kanan, Perak, Malaysia (4.3489° N, 100.8802° E) and stored at −20 °C in the Laboratory of Breeding Genetics at Universiti Putra Malaysia. The specimen is kept in the Depository Museum, Department of Aquaculture, Faculty of Agriculture, Universiti Putra Malaysia (Dr. Zafri Hassan, +60397694932, mzafri@upm.edu.my). The specimen has a voucher number of JAQ/BA/00001.

Total genomic DNA was extracted from approximately 2 mL of the blood sample following noninvasive procedures developed by Salleh and Esa (2022) and used for DNA extraction using the Qiagen Qiamp Minikit (Qiagen, Hilden, Germany) according to the manufacturer’s instructions. The DNA was sheared to 350 bp using a Bioruptor, followed by library preparation using the NEBUltra II Library Preparation Kit according to the manufacturer’s instructions. The constructed library was quantified with TapeStation (Agilent, Santa Clara, CA), followed by sequencing on a NovaSEQ6000 (Illumina, San Diego, CA) using a run configuration of 2 × 150 bp to generate approximately 5 GB of data. Raw paired-end reads were filtered with fastp (default setting) followed by de novo assembly using MEGAHIT v1.2.9. Identification of mitochondrial-derived contig(s) from the de novo assembly and mitogenome annotation used MitoZ v3.4.

## Results

**Figure 2.**
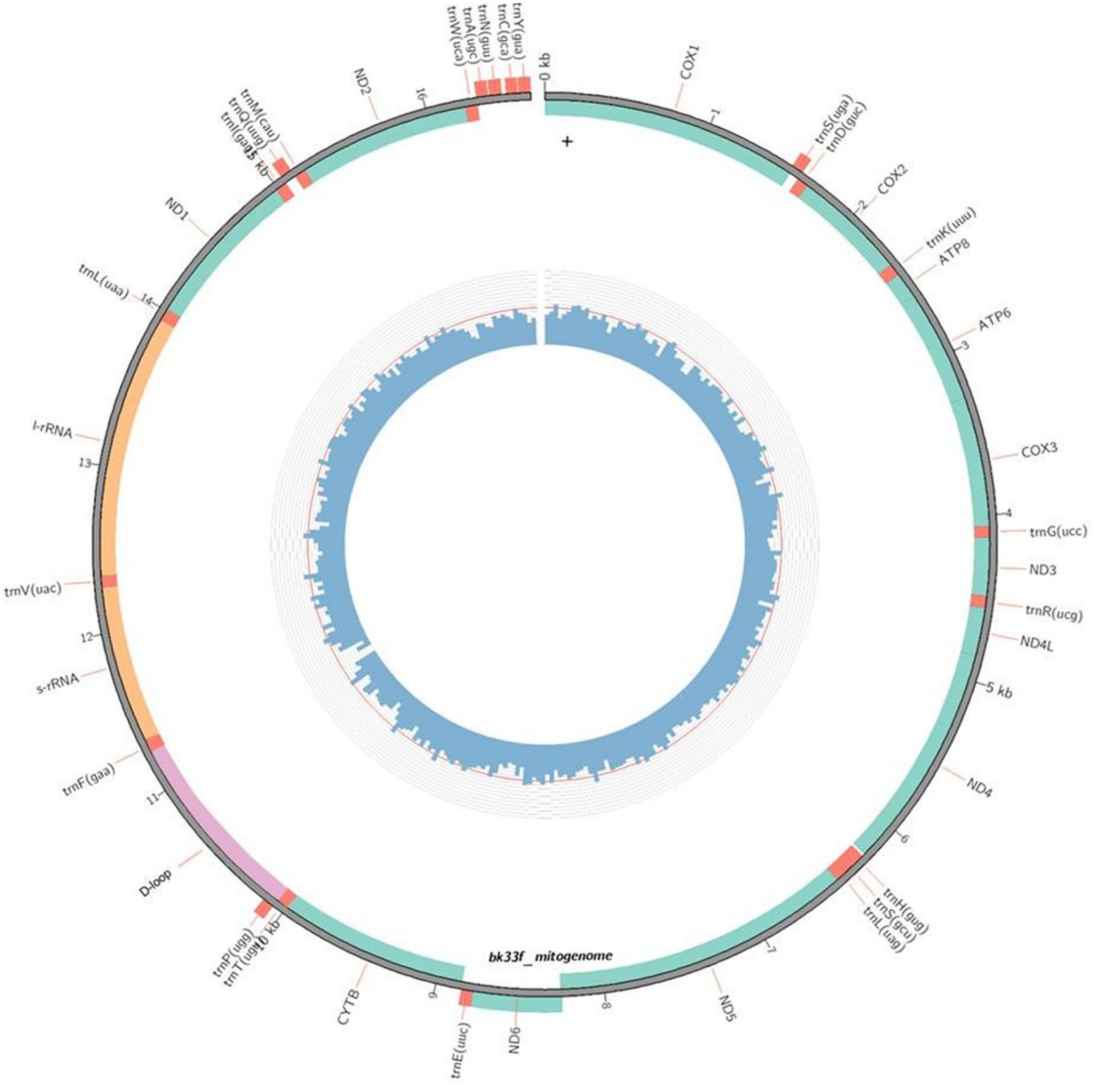
A novel genome map of the malaysian *B. affinis affinis* mitochondrial genome.

The first mitochondrial genome sequence from *B. affinis affinis* (specimen ID: BK33F) was submitted to GenBank and assigned the accession number OQ409915 (Figure 2). The assembled mitogenome length was 16,526 bp consisting of 13 conventional vertebrate protein-coding genes (PCGs), 22 transfer RNA (tRNA) genes, two ribosomal RNA (rRNA) genes, and a near-complete control region (D-loop) with no notable variations from the standard vertebrate mtDNA gene organization (Li et al. 2017; Tang et al. 2019).

## Discussion and conclusion

The mitogenome is composed of A (32.4%), T (25.3%), C (29.5%), and G (12.4%), in that order. A + T indicated a typical mitogenome sequence (61.0%). CO1 is the only protein-coding gene that begins with a start codon other than ATG. Most protein-coding genes are terminated by the stop codon TAA. Nine protein-coding genes end with complete stop codons (AGG, TAA, and TAG). Still, the remaining three genes end with T or TA as partial stop codons, which are presumed completed as TAA by post-transcriptional polyadenylation (Anderson et al. 1981). Except for the ND6 gene and eight tRNA genes, most *B. affinis affinis* mitochondrial genes are encoded on the H-strand. The trnCgca gene was the shortest among the mitochondrial protein-coding genes, while the ND5 gene was the longest. The 12S and 16S ribosomal RNAs have lengths of 965 and 1595 base pairs, respectively.

**Figure 3.**
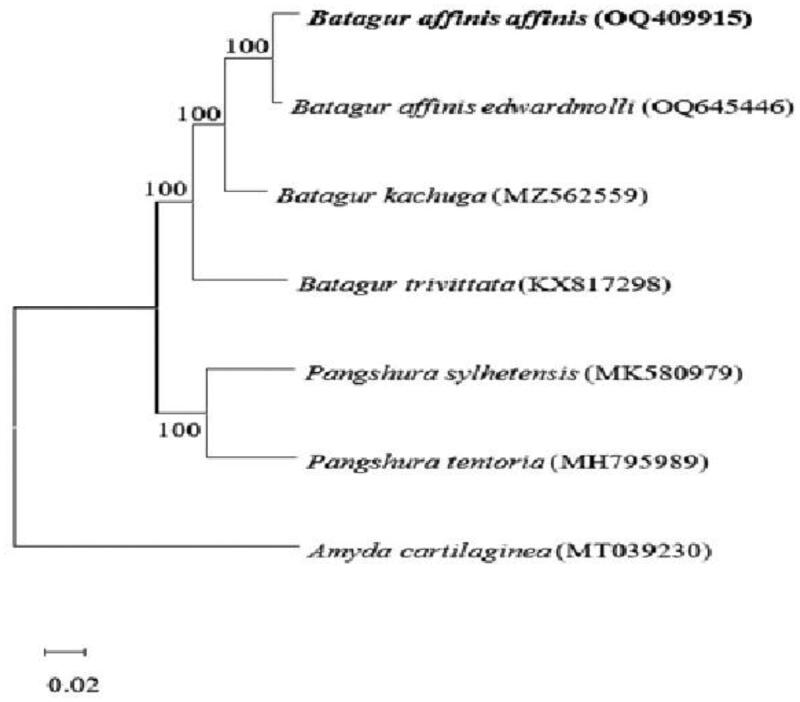
Neighbor-joining tree showing the genetic related of *Batagur* spp. The evolutionary history was inferred using the Neighbour-Joining method (NJ) (Saitou and Nei 1987). the bootstrap consensus tree inferred from 1000 replicates (Felsenstein 1985) is taken to represent the evolutionary history of the taxa analyzed (Felsenstein 1985). Branches corresponding to partitions reproduced in less than 50% of bootstrap replicates are collapsed. The percentage of replicate trees in which the associated taxa clustered together in the bootstrap test (1000 replicates) are shown next to the branches (Felsenstein 1985). the evolutionary distances were computed using the Kimura 2-parameter method (Kimura 1980) and are in the units of the number of base substitutions per site. This analysis involved four nucleotide sequences. Codon positions included were 1st + 2nd + 3rd + noncoding. All ambiguous positions were removed for each sequence pair (pairwise deletion option). there was a total of 11391 positions in the final dataset. Evolutionary analyses were conducted in MEGA X (Kumar et al. 2018). the following sequences were used: *Batagur affinis edwardmolli* OQ645446 (Salleh et al. 2023), *Batagur kachuga* MZ562559 (Das et al. 2021), *Batagur trivittata* KX817298 (Feng et al. 2017), *Pangshura sylhetensis* MK580979 (Kundu et al. 2020), *Pangshura tentoria* MH795989 (Kundu et al. 2019), and *Amyda cartilaginea* MT039230 (Cui et al. 2020).

Surprisingly, only two *Batagur* mitogenomes (*B. kachuga* and *B. trivittata*) have been reported in GenBank (https://www.ncbi.nlm.nih.gov/genbank) to date, despite the vast number of species in this genus. Figure 3 depicts the construction of a neighbor-joining (NJ) tree (Saitou and Nei 1987) using the dataset and MEGA X (Kumar et al. 2018). According to the cluster in the phylogenetic tree, *B. affinis affinis* and *B. kachuga* have the closest evolutionary ties. In contrast to the conventional CO1 or CytB partial gene sequence, the full mitogenome offers a much greater number of variable sites dramatic increase in the number of variable sites. It thus will be helpful in delineating the evolutionary relationship of *Batagur* spp. in the future.

## Data Availability

The genome sequence data supporting this study’s findings are openly available in GenBank of NCBI at https://www.ncbi.nlm.nih.gov/under the accession number OQ409915. The associated BioProject, SRA, and Bio-Sample numbers are PRJNA767629, SRR22444522, and SAMN31872828, respectively.
